# Using machine learning to predict anesthetic dose in fish: a case study using nutmeg oil

**DOI:** 10.3389/fvets.2025.1652115

**Published:** 2025-08-25

**Authors:** Mert Minaz, Cem Alparslan, Akif Er

**Affiliations:** ^1^Faculty of Fisheries, Recep Tayyip Erdogan University, Rize, Türkiye; ^2^Mechanical Engineering, Recep Tayyip Erdogan University, Rize, Türkiye

**Keywords:** *Myristica fragrans*, fish anesthesia, artificial neural network, hematological parameters, species sensitivity

## Abstract

Application of anesthetic chemicals in aquaculture is important to minimize stress under normal operations such as handling, transport, and artificial breeding. In the past decade, the preference for natural anesthetics over synthetic ones has increased due to welfare issues regarding fish welfare and food safety. This study investigates the anesthetic efficacy of nutmeg oil (*Myristica fragrans*) in three freshwater fish species—*Cyprinus carpio* (Common carp), *Acipenser gueldenstaedtii* (Danube sturgeon), and *Oncorhynchus mykiss* (Rainbow trout)—by modeling behavioral (Induction and recovery times) and hematological responses using artificial neural networks (ANNs). Experimental data obtained from previous studies were used to develop feed-forward ANN models for each species and parameter. Each model was trained using different activation functions (purelin, tansig, logsig) and optimization algorithms (traingda, trainrp, trains), and the optimal network architecture was selected based on prediction performance for each output variable. The ANN models successfully predicted species-specific responses, revealing distinct sensitivity levels to nutmeg oil. Model performance was assessed using R^2^, RMSE, and MAPE metrics, and the results revealed strong predictive capabilities of the ANN models across different fish species and physiological parameters. The most accurate models were obtained for WBC across all species, while induction and recovery times varied depending on fish physiology. The study demonstrates that ANN-based modeling can be a powerful tool for predicting optimal anesthetic doses and physiological responses without additional invasive testing. The results provide a scientific foundation for developing species-specific, welfare-limited anesthetic protocols and indicate the potential of artificial intelligence applications to experimental aquaculture practices.

## 1 Introduction

In aquaculture, anesthetic agents are routinely used to regulate the stress levels of aquatic organisms during operational procedures (1). Fish are subjected to various interventions such as stripping, transportation, and handling, all of which can induce significant stress responses (2–4). To mitigate these stress levels, fish are exposed to varying concentrations of anesthetics, ranging from mild sedation to deep anesthesia (5). Anesthetic agents function by interacting with the nervous system to induce temporary insensitivity to pain, thereby allowing medical or experimental procedures to be conducted without causing distress (6, 7).

The use of anesthetics in aquaculture dates back to the 1940s (8). Compared to other animals, the ability of fish to perceive pain was demonstrated relatively late (9). Consequently, the use of anesthetics in fish has evolved from merely calming stressed animals to becoming an ethical and scientific necessity in experimental studies (5). Numerous synthetic and natural anesthetics have been investigated for use in aquaculture (10). Natural anesthetic compounds have gained preference over synthetic ones due to their favorable physiological effects on fish and greater food and human safety through fish consumption (11–13). Within this context, nutmeg oil has been tested as an alternative anesthetic in various fish species (14–16). Native to the Maluku Islands of Indonesia, nutmeg oil is also distributed in India, Sri Lanka, South Africa, and the United States (17, 18). Its anesthetic properties are based on earlier findings using nutmeg powder (19), and prior narcotic reports have also described its use (20). The anesthetic effect of nutmeg is attributed to the compound myristicin, which is known to induce hallucinations, drowsiness, and tachycardia (21).

Depending on the intensity of the pain and the nature of the procedure, anesthesia is applied in three levels in fish: sedation, general anesthesia, and deep anesthesia (22, 23). The required depth and concentration of anesthesia depend on the nature of the procedure. For example, mild sedation may be sufficient for short-term handling, while invasive procedures typically require deeper anesthesia with higher anesthetic concentrations (9). Determining the appropriate concentration of an anesthetic agent is critical not only for cost-effectiveness but also to ensure that the aquatic organism reaches the desired anesthetic state. Historically, optimal anesthetic concentrations and stages were determined based on basic behavioral and physiological responses in fish, such as equilibrium loss, swimming patterns, opercular movements, and heart rate (24, 25). However, in modern aquaculture, the determination of optimal anesthetic concentrations involves a broad range of parameters, including hematological, histological, biochemical, and behavioral indicators (26, 27).

The effective concentration of an anesthetic agent varies widely among fish species and is influenced by multiple factors, including fat content, hypoxia sensitivity, size class, stress level, and water quality parameters such as dissolved oxygen (3, 28–30). Therefore, species-specific concentration ranges must be established. While numerous studies have investigated these thresholds, the use of machine learning and artificial neural networks (ANN) to predict anesthetic concentrations remains an unexplored approach in this field.

ANNs are effective prediction tools widely utilized in industrial operations to model complex relationships between input and output parameters. They are built using experimental data and preset parameters and can be employed for prediction under untapped conditions. Especially useful where the conventional experimental methods could not model process behavior, ANN models are effective and safe substitutes (31–33). ANNs are structured as multilayered networks made up of an input layer, one or more hidden layers, and an output layer. The input and output layers are made of neurons that represent independent and dependent variables, respectively, while the hidden layers process the data by acquiring the relationships among these neurons. While theoretically there is no limit to the number of hidden layers, one or two are often sufficient and effective in practice (34, 35). The output layer calculates the data from the input taken by the hidden layers to give the final output vector. Research from literature indicates that ANN models with a single-output variable typically tend to have higher accuracy than multi-output setups. All neurons in the network are connected to other neurons with adjustable weights, which are optimized during training so that the error function is minimal. With a proper training algorithm, ANN models can be tailored to make highly accurate predictions for a specific problem. While applications of ANN are well researched in most domains, their application to aquaculture constitutes significant research potential.

In this context, the present study aims to comparatively evaluate the hematological responses and anesthetic efficacy of nutmeg oil in three freshwater fish species—common carp (*Cyprinus carpio*), Danube sturgeon (*Acipenser gueldenstaedtii*), and rainbow trout (*Oncorhynchus mykiss*)—using ANN models to analyze interspecies sensitivity differences. The findings of this research contribute not only to the fields of experimental biology and aquatic animal health, but also to interdisciplinary domains such as computational modeling and predictive management of aquatic organisms. Ultimately, the study aims to establish a scientific basis for the development of safer anesthetic protocols that prioritize animal welfare. To the best of our knowledge, this is the first study to integrate hematological and behavioral anesthetic data into species-specific artificial neural network (ANN) models across three freshwater fish species. This novel approach enables predictive assessment of anesthetic effects and sets a foundation for intelligent sedation management in aquaculture.

## 2 Materials and methods

### 2.1 Ethical statement

No new animal experiments were performed in this study. The datasets used were sourced from previously published studies by our research group (14–16), each of which received ethical approval from the Ethical Committee of Recep Tayyip Erdogan University, Türkiye (Decision No: 2024/05) and adhered to the relevant guidelines for fish husbandry and welfare.

### 2.2 Extraction of raw data

The present study was designed based on the outcome data of three previous experimental investigations conducted by our research team (14–16). These studies focused on evaluating the anesthetic effects of nutmeg oil in common carp (15), Danube sturgeon (14), and rainbow trout (16). Shared parameters across all three studies—including induction time (IT), recovery time (RT), white blood cell count (WBC), red blood cell count (RBC), hemoglobin concentration (HGB), and hematocrit level (HCT)—were selected as the output variables in the current model. Each fish species (ten individuals) was exposed to three different concentrations of nutmeg oil: 800 μL/L, 1,200 μL/L, and 1,400 μL/L for common carp; 500 μL/L, 750 μL/L, and 1,000 μL/L for Danube sturgeon; and 400 μL/L, 600 μL/L, and 800 μL/L for rainbow trout based on previously reported effective ranges providing induction times under 3 min and recovery times under 5 min (1). Induction time was determined by observing key behavioral indicators such as complete unresponsiveness to stimuli, abnormal opercular movements, and loss of equilibrium (36). Fish behavior under anesthesia was monitored for a duration of 7 min, which allowed sufficient time to observe both rapid and delayed anesthetic responses while minimizing prolonged exposure to handling stress. Recovery was defined as the moment when the fish resumed pre-anesthesia behavioral patterns, including active swimming and clear response to external tactile or visual stimuli, as observed under clean, controlled water conditions. Randomly selected fish were subjected to terminal blood sampling following percussion stunning, ensuring immediate unconsciousness and humane euthanasia prior to sample collection. Blood samples were collected from the caudal vein with a 2-mL syringe and quickly transferred to EDTA K3 tubes. The hematological parameters were measured using an automated hematology analyzer (Prokan-6800VET) (37).

### 2.3 Design of artificial neural network (ANN)

**Figure 1:**
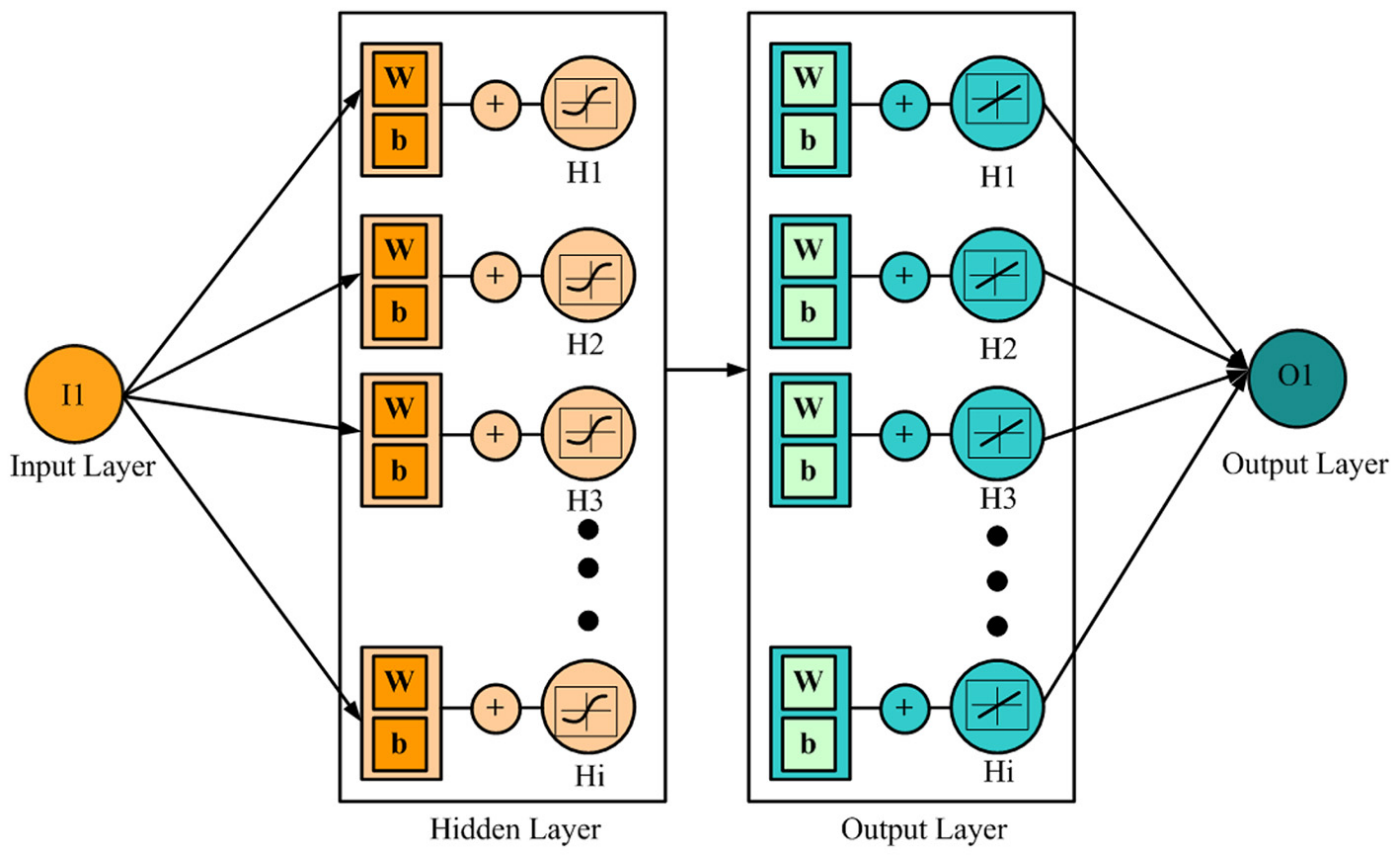
The architecture of the neural network. I1, input neuron; w, weight; b, bias; O1, output neuron.

In the present study, ANN modeling was performed using a subprogram within MATLAB (R19a) to analyze the anesthetic efficacy of nutmeg oil in common carp, Danube sturgeon, and rainbow trout. The modeling focused on the output variables, including induction time (IT), recovery time (RT), and hematological parameters such as WBC, RBC, HGB, and HCT. The ANN modeling process consisted of two stages: training and testing. During the training phase, the input and output values provided to the network were carefully controlled to minimize error. In the testing phase, the network weights were kept constant, and the model was used to predict outcomes based on new input values. The output values were analyzed individually using a generalized feed-forward network structure. In the model, concentration (C) was defined as the input parameter, while IT, RT, WBC, RBC, HGB, and HCT were designated as output parameters. For each fish species, a total of 30 data points (10 individuals × 3 concentrations) were obtained. Of these, 18 were used for training and 12 for testing the ANN models. Input and output data were imported into the software, and optimal network architectures were determined through multiple experimental trials using these datasets (Figure 1). Detailed architectural parameters (e.g., number of layers, neurons per layer, activation functions, and optimizers) varied by species and were selected based on model performance. These optimal configurations are presented in the Results section. Optimal network architectures were determined through a systematic trial-and-error process, where multiple combinations of hidden neurons, activation functions, and training algorithms were tested. For each configuration, the coefficient of determination (*R*^2^) on the test dataset was used to evaluate performance, and the model with the highest *R*^2^ and lowest overfitting was selected as optimal.

Due to limited sample size, cross-validation was not performed. Prior to model training, all input and output data were normalized to the range of 0 to 1 using min-max normalization to ensure equal weighting and improve training stability. Furthermore, cross-validation techniques such as k-fold or bootstrapping were not applied, as further splitting the data could compromise model training. Instead, model performance was evaluated using an independent test dataset, and simple architectures were preferred to reduce the risk of overfitting.

During the training and testing process of the network, model performance was measured by Root Mean Square Error (RMSE; Equation 1), while the evaluation of neural network model results was done by Mean Absolute Percentage Error (MAPE; Equation 2; N: number of data). The following criteria were taken into account for MAPE values in the evaluation of the model: MAPE ≤ 10%: High accuracy, 10% < MAPE ≤ 20%: Good, 20% < MAPE < 50%: Acceptable and MAPE >50%: Misleading prediction. The statistical significance and accuracy of the developed models were determined by the R' correlation coefficient (Equation 3).


(1) $$\begin{array}{l} RMSE=\sqrt{\frac{1}{N}\sum_{n=1}^{N}{\left(actual-predicted\right)}^{2}} \end{array}$$

(2) $$\begin{array}{l} MAPE=\frac{1}{N}\sum_{n=1}^{N}\left(\frac{\left|actual-predicted\right|}{actual}\right)\times 100 \end{array}$$

(3) $$\begin{array}{l} R^{2}=1-\left(\frac{\sum_{n=1}^{N}{\left(actual-predicted\right)}^{2}}{\sum_{n=1}^{N}{\left(predicted\right)}^{2}}\right) \end{array}$$

## 3 Results

In this study, various ANN-based approaches were developed to model the efficacy of nutmeg oil—specifically in terms of IT, RT, and hematological outputs (WBC, RBC, HGB, and HCT)—in common carp, Danube sturgeon, and rainbow trout. For the prediction of each parameter (IT, RT, WBC, RBC, HGB, HCT), separate ANN models were constructed, each incorporating a distinct combination of activation functions and learning algorithms. The most suitable network architecture was determined individually for each parameter, and the corresponding input-hidden-output layer structures along with model parameters (weights and bias values) are summarized. The activation functions employed in the models varied and included linear (purelin), logarithmic sigmoid (logsig), and hyperbolic tangent sigmoid (tansig). Each function was selected to facilitate linear and non-linear transformations within the network in order to enhance the learning capability of the model.

### 3.1 The ANN model for common carp

For common carp, the learning algorithms employed included Gradient Descent with Adaptive Learning Rate (traingda), TRAIN Resilient Backpropagation (trainrp), Gradient Descent with Adaptive Learning Rate Backpropagation (trainsgd), and Sequential Order Incremental Training with Learning Functions (trains; Table 1). These algorithms functioned by updating the network weights to minimize the error function, thereby enabling the effective training of the models (Table 2). For the parameters IT and RT, the models utilized purelin-purelin activation functions and were trained using the traingda algorithm. The optimal network architecture for both parameters was determined to be 1-10-1, where the input data was processed through 10 neurons in the hidden layer and then passed to the output layer.

**Table 1:** Activation functions (Purelin, Tansig, Logsig) used in artificial neural network (ANN) models developed for common carp, including the position of each prediction parameter within the network structure (Input, Output, Input-Output) and the output layer calculation formula.

	**Purelin**	**Tansig**	**Logsig**	**Target**
	*F*_*i*_ = *C*.*W*_*MFi*_+θ*i*	$F_{i}=\frac{2}{1+\;e^{-2\left[C.W_{MFi}+\theta i\right]}}-1$	$F_{i}=\frac{2}{1+\;e^{-\left[C.W_{MFi}+\theta i\right]}}$	
IT	Input-output			$\overset{n}{\underset{i=1}{\sum }}F_{i}.L_{w}+\theta_{j}$
RT	Input-output				
WBC	Input-output				
RBC	Output	Input			
HGB	Output		Input		
HCT	Output	Input			

Where, C: the nutmeg oil concentration; W_MFi_: the weight from the input layer to the hidden layer; θ_i_: the bias of the hidden layer; F_i_: the output of the activation function in the hidden layer; L_w_: the weight from the hidden layer to the output layer; and θ_j_: the bias of the output layer.

**Table 2:** Weight (W), bias (θ), and activation function values used in artificial neural network (ANN) models for IT, RT, WBC, RBC, HGB, and HCT parameters in common carp.

**IT**	***i***	**W_1_**	**θ_i_**	**L_w_**	**θ_11_**		***i***	**W_1_**	**θ_i_**	**L_w_**	**θ_7_**
	1	−0.8556	−0.2698	0.7519		WBC	1	−0.6185	−9.9570	−0.7955	
	2	0.1007	0.5011	−0.8926			2	−0.3684	−9.8602	−0.2502	
	3	−0.2973	−0.9577	−0.3949			3	−0.4067	−10.1663	−0.2883	10.3895
	4	0.2712	0.7431	0.4685			4	−0.4594	10.5147	0.7288	
	5	0.3896	0.4715	−0.1145	0.0162		5	0.9264	−9.6797	−0.4368	
	6	0.7886	0.3759	0.5205			6	−0.1384	−9.6860	−0.2894	
	7	−0.6954	0.1873	−0.7558							
	8	−0.9027	0.3302	0.9311							
	9	0.9690	0.0909	0.1494							
	10	0.8942	0.1499	0.5051							
	***i***	**W**_1_	θ_i_	**L**_w_	θ_11_		***i***	**W**_1_	θ_i_	**L**_w_	θ_8_
	1	−0.2513	55.9997	−12.1470	13.8936	RBC	1	0.4099	39.2004	0.7834	−0.0054
	2	0.0288	−52.8889	−13.6276			2	−0.0410	35.9333	−1.4201	
	3	0.0053	−49.7778	−10.2792			3	−0.0437	32.6666	1.0657	
	4	0.0262	−46.6666	−10.7937			4	0.2456	−29.3997	0.6414	
	5	−0.2455	−43.5558	−13.3260			5	−0.2320	−26.1336	−1.5920	
	6	−0.0704	40.4444	−14.6945			6	−0.0380	22.8666	−0.2820	
	7	−0.2121	−37.3336	−13.9904			7	−0.0627	19.5999	1.0064	
	8	0.0606	−34.2222	12.8529							
	9	−0.1183	31.1110	−11.2552							
	10	0.0108	−28.0000	−8.22270							
**HGB**	***i***	**W**_1_	θ_i_	**L**_w_	θ_6_		***i***	**W**_1_	θ_i_	**L**_w_	θ_8_
	1	−5.6239	55.9944	−1.973	13.8936	RBC	1	11.7392	44.8117	0.6057	0.0280
	2	0.0375	−49.0000	−0.9873			2	−0.2481	−41.6002	−2.2306	
	3	0.4596	−41.9994	0.1942			3	−0.0450	38.3999	−0.6716	
	4	−0.0984	34.9999	−4.3736			4	−0.0125	−35.2000	−0.4986	
	5	−0.9659	−28.0020	−2.6583			5	0.1577	−31.9998	1.0191	
							6	0.0446	−28.8000	−0.4964	
							7	0.0518	−25.5999	0.3058	
							8	2.3678	−22.3953	0.6555	

For each parameter, the optimal network architecture and the values of L_w_ ve θ_output_ used in the corresponding mathematical modeling process are also provided. IT, Induction Time; RT, Recovery Time; WBC, White Blood Cell count; RBC, Red Blood Cell count; HGB, Hemoglobin concentration; HCT, Hematocrit level.

For WBC, the ANN model was configured with purelin-purelin activation functions and trained using the trainrp algorithm. A relatively simpler 1-6-1 architecture was selected for this model, which was particularly well-suited to capturing the linear distribution patterns of white blood cells. For RBC prediction, a tansig activation function was employed in the hidden layer and purelin in the output layer, with the training process conducted via the trainsgd algorithm. The optimal architecture was determined to be 1-7-1, allowing for effective modeling of non-linear relationships through the use of the tansig function.

In the case of HGB, a logsig-purelin combination was used, and the model was trained using the trains algorithm. The best-performing architecture for this parameter was 1-5-1, with the logsig function facilitating the modeling of data exhibiting saturation tendencies. Finally, for HCT, the model used tansig-purelin activation functions and was trained with the traingda algorithm. Similar to the RBC model, the non-linear relationships were successfully captured using the tansig function, and the optimal network structure was also identified as 1-7-1.

**Figure 2:**
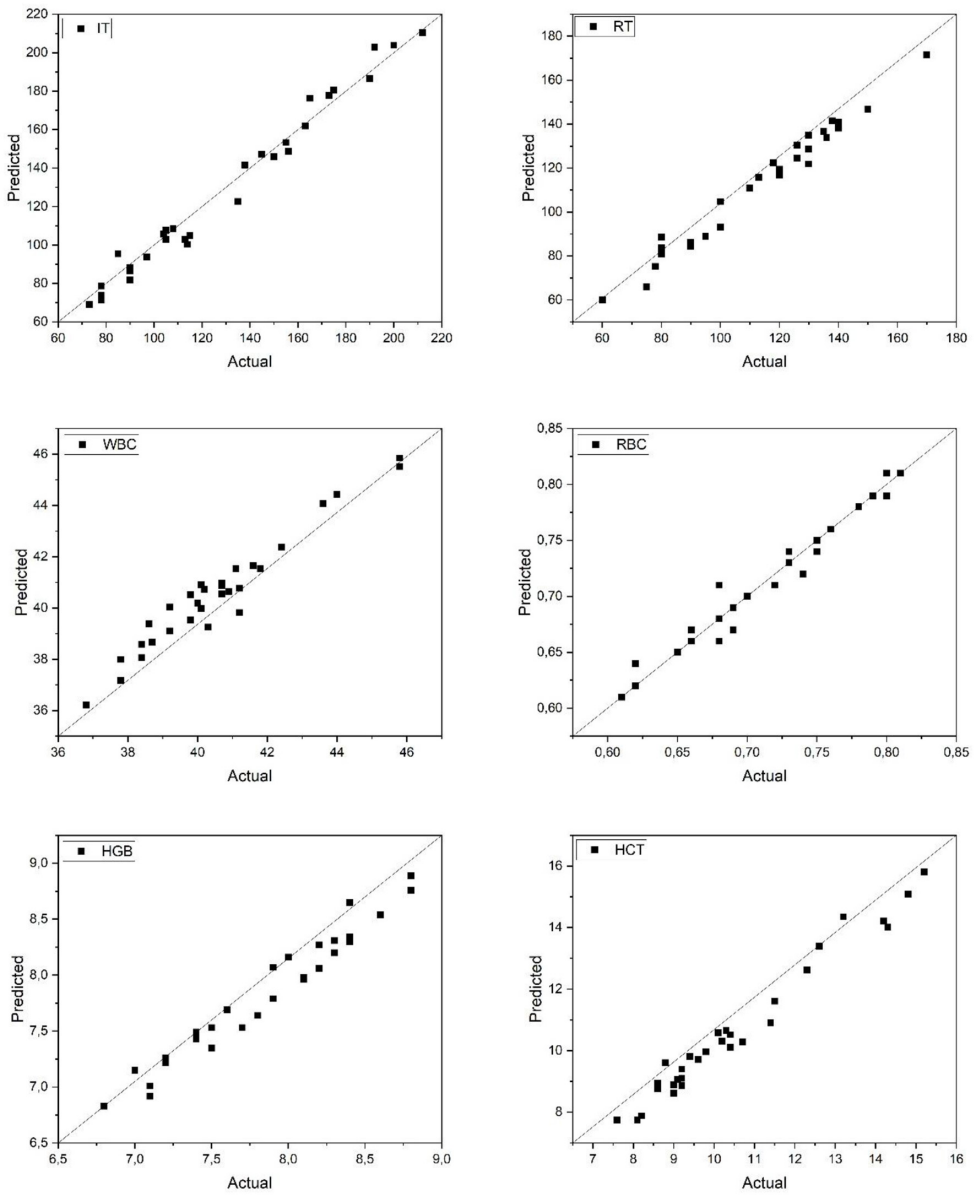
Predicted and actual values of IT, RT, WBC, RBC, HGB, and HCT for common carp. IT, Induction Time; RT, Recovery Time; WBC, White Blood Cell count; RBC, Red Blood Cell count; HGB, Hemoglobin concentration; HCT, Hematocrit level.

Figure 2 illustrates the relationship between actual and predicted values for six physiological parameters (IT, RT, WBC, RBC, HGB, and HCT) in Common carp as estimated by the artificial neural network (ANN) model. The data points align closely with the 1:1 diagonal line, indicating high predictive accuracy. The IT and RT parameters, representing anesthesia induction and recovery times, show particularly strong correlation with the observed values, suggesting that the model effectively captured the dynamic response patterns to anesthetic exposure. Hematological parameters (WBC, RBC, HGB, and HCT) also demonstrated high consistency between actual and predicted values, further supporting the robustness of the model across diverse physiological indices. These results validate the ANN's capacity to reliably estimate biological responses in Common carp based on input features, enhancing its applicability for precision monitoring in aquaculture practices.

### 3.2 The ANN model for Danube sturgeon

For the Danube sturgeon, the learning algorithms applied included Adaptive Learning Rate Gradient Descent (traingda), Gradient Descent with Adaptive Learning Rate Backpropagation (also traingda), and Sequential Order Incremental Training with Learning Functions (trains; Table 3). These algorithms aimed to enhance model performance by optimizing weight updates through error backpropagation (Table 4). For the IT parameter, a logsig-purelin activation function combination was used along with the traingda algorithm, resulting in an optimal network architecture of 1-10-1. This model was structured to predict the initial duration of the anesthetic's effect on the organism.

**Table 3:** Activation functions (Purelin, Tansig, Logsig) used in artificial neural network (ANN) models developed for Danube sturgeon, including the position of each prediction parameter within the network structure (Input, Output, Input-Output) and the output layer calculation formula.

	**Purelin**	**Tansig**	**Logsig**	**Target**
	*F*_*i*_ = *C*.*W*_*MFi*_+θ*i*	$F_{i}=\frac{2}{1+\;e^{-2\left[C.W_{MFi}+\theta i\right]}}-1$	$F_{i}=\frac{2}{1+\;e^{-\left[C.W_{MFi}+\theta i\right]}}$	
IT	Output		Input	$\overset{n}{\underset{i=1}{\sum }}F_{i}.L_{w}+\theta_{j}$
RT	Output	Input			
WBC	Input-output				
RBC	Output	Input			
HGB	Input-output				
HCT	Output	Input			

Where, C: the nutmeg oil concentration; W_MFi_: the weight from the input layer to the hidden layer; θ_i_: the bias of the hidden layer; F_i_: the output of the activation function in the hidden layer; L_w_: the weight from the hidden layer to the output layer; and θ_j_: the bias of the output layer.

**Table 4:** Weight (W), bias (θ), and activation function values used in artificial neural network (ANN) models for IT, RT, WBC, RBC, HGB, and HCT parameters in Danube sturgeon.

**IT**	***i***	**W_1_**	**θ_i_**	**L_w_**	**θ_11_**		***i***	**W_1_**	**θ_i_**	**L_w_**	**θ_8_**
	1	−2718.79	124.454	33.836	87.513	WBC	1	−0.417	−12.954	−0.597	11.457
	2	14.3672	118.232	77.317			2	−0.266	−11.870	−0.151	
	3	12.351	113.683	77.251			3	−0.501	−14.162	−0.484	
	4	2020.48	105.781	72.113			4	−0.352	17.524	0.628	
	5	4.929	−99.5560	66.399			5	0.827	−19.674	−0.314	
	6	0.084	−93.3330	−152.419			6	−0.236	−4.6810	−0.758	
	7	0.206	87.111	−149.968			7	−0.452	−4.5260	−0.444	
	8	−0.03700	−80.9220	67.11							
	9	−27.0320	−77.8730	19.807							
	10	−2565.75	124.454	29.482							
**RT**	***i***	**W**_1_	θ_i_	**L**_w_	θ_9_		***i***	**W**_1_	θ_i_	**L**_w_	θ_6_
	1	0.114	104.533	7.639	50.091	RBC	1	0.225	29.212	0.568	−0.0156
	2	0.056	−98.1330	−0.4640			2	−0.011	34.523	−1.254	
	3	0.051	−91.7330	−0.4660			3	−0.045	31.562	1.125	
	4	−0.043	85.333	50.103			4	0.125	−25.548	0.684	
	5	−0.039	78.933	28.969			5	−0.454	−24.223	−1.875	
	6	−0.032	72.533	29.612							
	7	−0.054	66.312	−94.459							
	8	−0.025	59.733	47.775							
**HGB**	***i***	**W**_1_	θ_i_	**L**_w_	θ_8_		***i***	**W**_1_	θ_i_	**L**_w_	θ_11_
	1	−5.623	55.994	−1.973	4.964	RBC	1	10.215	34.562	0.665	0.019
	2	0.037	−49.000	−0.987			2	−0.2450	−41.325	−2.624	
	3	0.459	−41.999	0.194			3	−0.0860	36.982	−0.623	
	4	−0.098	34.999	−4.373			4	−0.0170	−32.215	−0.456	
	5	−0.965	−28.002	−2.658			5	0.167	−32.953	1.212	
	6	−0.875	−21.456	−1.256			6	0.047	−22.646	−0.565	
	7	−1.255	31.256	−1.287			7	0.051	−23.456	0.654	
							8	0.658	−23.565	0.456	
							9	0.845	−21.654	0.962	
							10	2.475	−22.395	0.665	

For each parameter, the optimal network architecture and the values of L_w_ ve θ_output_ used in the corresponding mathematical modeling process are also provided. IT, Induction Time; RT, Recovery Time; WBC, White Blood Cell count; RBC, Red Blood Cell count; HGB, Hemoglobin concentration; HCT, Hematocrit level.

For RT, a model employing tansig-purelin activation functions trained with the traingda algorithm was implemented. A 1-8-1 architecture, containing eight neurons in the hidden layer, provided effective results in estimating recovery duration following anesthesia. For WBC, the ANN model was developed using purelin-purelin activation functions and trained via the traingda algorithm. The optimal architecture was identified as 1-7-1, effectively representing the linear relationship between input and output variables.

In modeling RBC, considering the non-linear nature of the data, tansig-purelin activation functions were chosen, and the model was trained using traingda. The optimal ANN architecture for this parameter was determined as 1-5-1. For HGB, a model utilizing purelin-purelin activation functions trained with the trains algorithm yielded the best performance, with a 1-7-1 network structure adequately capturing hemoglobin concentration variability. For HCT, the model was developed using tansig-purelin activation functions and the traingda algorithm. A 1-10-1 architecture was selected to provide a higher representation capacity for this parameter.

**Figure 3:**
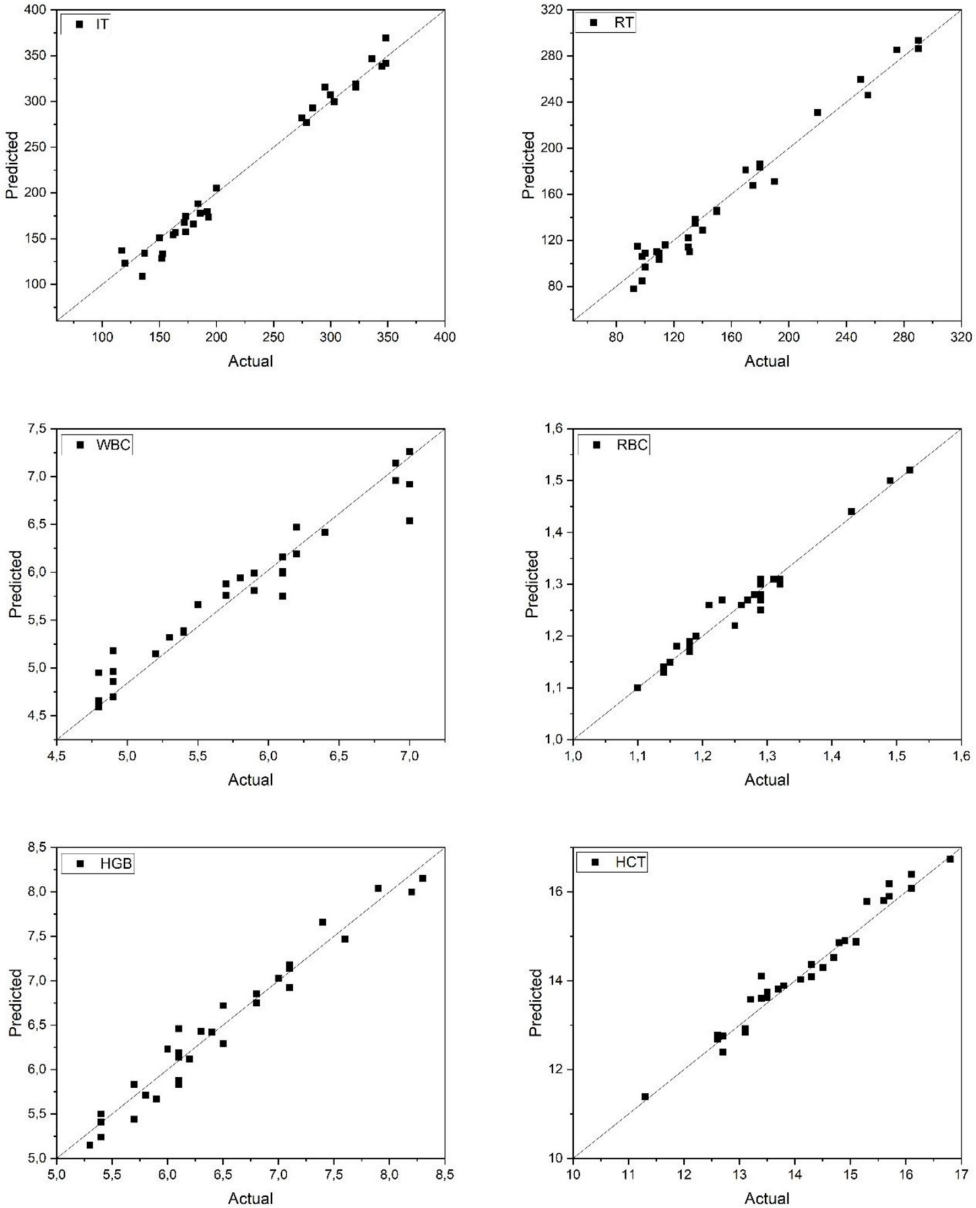
Predicted and actual values of IT, RT, WBC, RBC, HGB, and HCT for danube sturgeon. IT, Induction Time; RT, Recovery Time; WBC, White Blood Cell count; RBC, Red Blood Cell count;HGB, Hemoglobin concentration; HCT, Hematocrit level.

Figure 3 presents the comparison between actual and predicted values for IT, RT, and hematological parameters (WBC, RBC, HGB, and HCT) in Danube sturgeon using the ANN model. The scatter plots show a strong alignment of predicted values along the ideal 1:1 line, indicating high model accuracy. The ANN accurately captured the variation in both anesthesia-related parameters and hematological indices, with minimal dispersion around the regression line. In particular, WBC and RBC counts exhibited tight clustering of data points, suggesting precise model performance in estimating leukocyte and erythrocyte levels. Furthermore, IT and RT predictions demonstrated high consistency with observed values, reflecting the model's effectiveness in tracking temporal physiological responses. These findings underscore the model's capacity to generalize well across parameters and affirm its suitability for predictive monitoring in sturgeon species within aquaculture systems.

### 3.3 The ANN model for rainbow trout

ANN modeling was performed using two learning algorithms: Adaptive Learning Rate Gradient Descent (traingda) and Sequential Order Incremental Training (trains) for the rainbow trout (Table 5). These algorithms aimed to improve prediction performance by updating the network weights based on error backpropagation (Table 6). For the IT parameter, a model incorporating tansig-purelin activation functions and trained using the trains algorithm was implemented. The optimal network architecture was identified as 1-8-1, and the model was structured to estimate the time required for the fish's initial physiological response to anesthesia.

**Table 5:** Activation functions (Purelin, Tansig, Logsig) used in artificial neural network (ANN) models developed for rainbow trout, including the position of each prediction parameter within the network structure (Input, Output, Input-Output) and the output layer calculation formula.

	**Purelin**	**Tansig**	**Logsig**	**Target**
	*F*_*i*_ = *C*.*W*_*MFi*_+θ*i*	$F_{i}=\frac{2}{1+\;e^{-2\left[C.W_{MFi}+\theta i\right]}}-1$	$F_{i}=\frac{2}{1+\;e^{-\left[C.W_{MFi}+\theta i\right]}}$	
IT	Output	Input		$\overset{n}{\underset{i=1}{\sum }}F_{i}.L_{w}+\theta_{j}$
RT	Output	Input			
WBC	Input-output				
RBC	Output		Input		
HGB	Output	Input			
HCT	Output		Input		

Where, C: the nutmeg oil concentration; W_MFi_: the weight from the input layer to the hidden layer; θ_i_: the bias of the hidden layer; F_i_: the output of the activation function in the hidden layer; L_w_: the weight from the hidden layer to the output layer; and θ_j_: the bias of the output layer.

**Table 6:** Weight (W), bias (θ), and activation function values used in artificial neural network (ANN) models for IT, RT, WBC, RBC, HGB, and HCT parameters in rainbow trout.

**IT**	***i***	**W_1_**	**θ_i_**	**L_w_**	**θ_9_**		***i***	**W_1_**	**θ_i_**	**L_w_**	**θ_6_**
	1	−40.259	−52.295	−2.814	3.344	WBC	1	0.211	0.91	−0.968	0.883
	2	3.233	49.069	2.717			2	−0.322	0.048	0.684	
	4	26.072	42.68	1.019			3	−0.834	0.562	−0.151	
	5	0.092	−39.466	0.075			4	−0.050	−0.924	0.813	
	6	0.0428	−36.266	−0.199			5	0.556	−0.724	0.702	
	7	−6.2110	−33.074	−1.122							
	8	958.68	31.065	−0.792							
**RT**	***i***	**W**_1_	θ_i_	**L**_w_	θ_7_		***i***	**W**_1_	θ_i_	**L**_w_	θ_5_
	1	0.019	−39.200	−28.329	27.481	RBC	1	−0.022	22.399	−0.816	−0.642
	2	−0.029	35.857	−61.160			2	0.063	−18.666	0.488	
	3	−0.055	32.48	−28.213			3	0.104	−14.933	1.034	
	4	−0.012	29.12	−28.623			4	0.012	−11.200	−0.651	
	5	−0.027	−25.760	−28.335							
	6	−0.004	22.4	28.681							
**HGB**	***i***	**W**_1_	θ_i_	**L**_w_	Θ_11_		***i***	**W**_1_	θ_i_	**L**_w_	θ_9_
	1	−1.097	−56.001	−0.219	0.064	RBC	1	11.739	44.811	0.605	0.028
	2	0.086	−52.888	0.481			2	−0.248	−41.600	−2.237	
	3	−0.055	49.777	0.488			3	−0.0450	38.399	−0.672	
	4	−0.054	46.666	0.17			4	−0.012	−35.200	−0.498	
	5	−2.907	−43.559	−0.567			5	0.157	−31.999	1.019	
	6	−1.862	−40.447	−0.299			6	0.044	−28.800	−0.496	
	7	−0.063	37.333	−0.657			7	0.051	−25.599	0.305	
	8	0.055	−34.222	−0.404			8	2.367	−22.395	0.655	
	9	0.055	−34.222	−0.404							
	10	−2.733	−28.005	−0.438							

For each parameter, the optimal network architecture and the values of L_w_ ve θ_output_ used in the corresponding mathematical modeling process are also provided. IT, Induction Time; RT, Recovery Time; WBC, White Blood Cell count; RBC, Red Blood Cell count; HGB, Hemoglobin concentration; HCT, Hematocrit level.

For the RT parameter, tansig-purelin activation functions were again employed, but this time trained using the traingda algorithm. The model, which included six neurons in the hidden layer, utilized a 1-6-1 architecture and successfully predicted recovery time. For the WBC parameter, the model used purelin-purelin activation functions in combination with the traingda algorithm. A linear 1-5-1 architecture effectively captured the relationship between input and output data.

In modeling RBC, due to the non-linear nature of the data distribution, logsig-purelin activation functions were used, and the model was trained with traingda. The optimal architecture for this parameter was determined to be 1-4-1. For HGB, tansig and purelin activation functions were used in the hidden and output layers, respectively, with the training process carried out using the trains algorithm. The 1-10-1 structure yielded satisfactory performance in predicting hemoglobin concentration. Finally, the HCT model was developed using logsig-purelin activation functions and trained with traingda. The relationship between input and output data was successfully modeled using a 1-8-1 network architecture.

**Figure 4:**
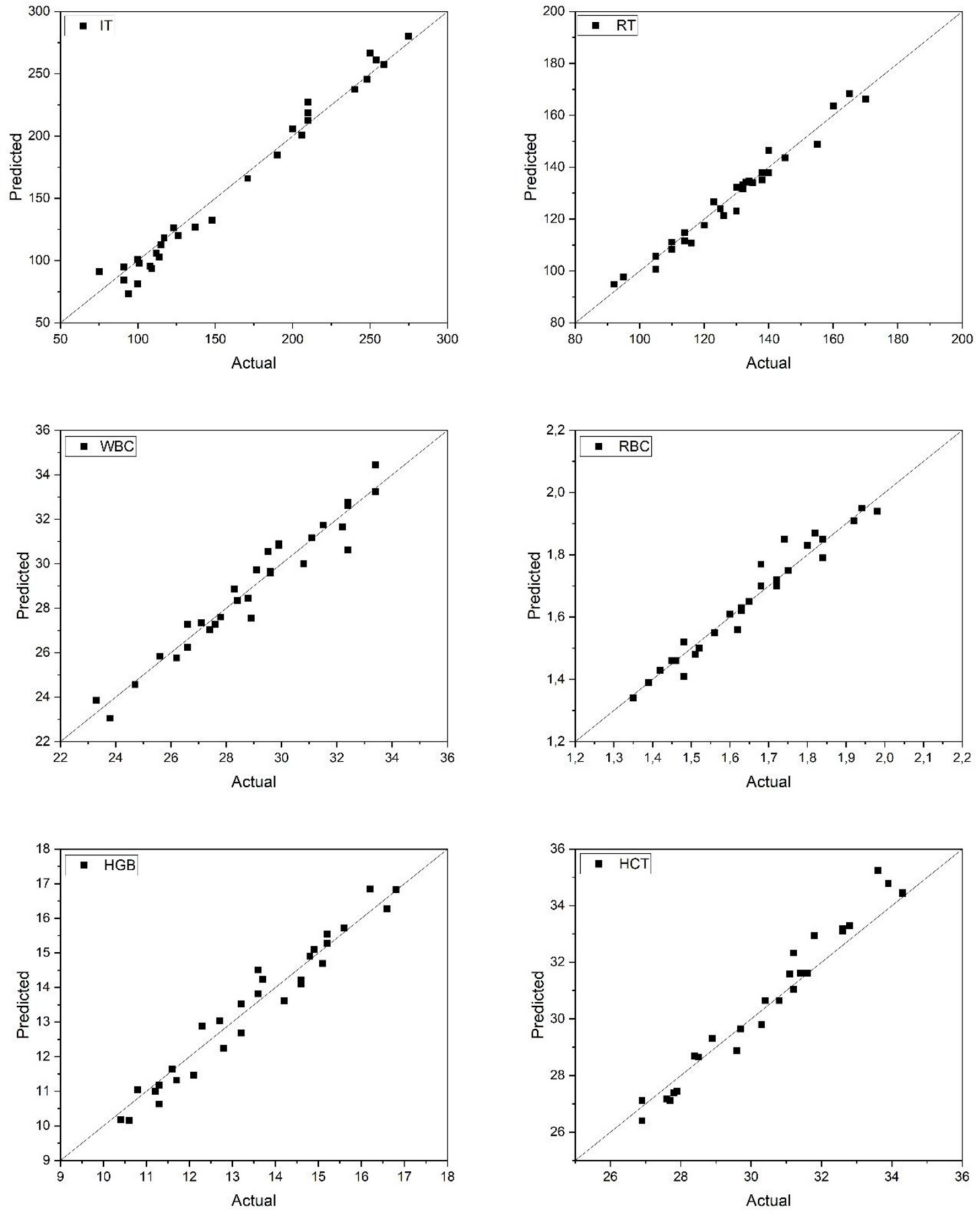
Predicted and actual values of IT, RT, WBC, RBC, HGB, and HCT for rainbow trout. IT, Induction Time; RT, Recovery Time; WBC, White Blood Cell count; RBC, Red Blood Cell count; HGB, Hemoglobin concentration; HCT, Hematocrit level.

Figure 4 displays the predicted vs. actual values for anesthesia-related (IT, RT) and hematological (WBC, RBC, HGB, HCT) parameters in Rainbow trout. The ANN model achieved strong predictive alignment across all parameters, with data points clustering near the ideal diagonal line. Particularly, the RBC and WBC predictions exhibited minimal deviation, reflecting stable model performance. Overall, the network provided accurate estimations of both physiological and hematological responses, reinforcing its practical value in trout welfare monitoring under anesthetic exposure.

### 3.4 Cross-species predictive modeling results using artificial neural networks

Table 7 presents the performance evaluation of the artificial neural network (ANN) models developed to predict various hematological (WBC, RBC, HGB, HCT) and anesthesia-related (IT, RT) parameters in Common carp, Danube sturgeon, and Rainbow trout. The models exhibited high predictive accuracy across both training and testing datasets, with *R*^2^ values generally exceeding 0.92 in most cases. Notably, the training phase yielded slightly better performance metrics (lower RMSE and MAPE) compared to the testing phase, indicating reliable model generalization without significant overfitting.

**Table 7:** Performance metrics (RMSE, MAPE, and *R*^2^) of the artificial neural network (ANN) model in predicting hematological and anesthesia-related parameters across three fish species (Common carp, Danube sturgeon, and Rainbow trout) during training and testing phases.

**Species**	**Parameter**	**Type**	**RMSE**	**MAPE (%)**	***R* ^2^**
Common carp	IT	Training	0.14	3.58	0.9705
		Testing	0.26	5.06	0.9331
	RT	Training	0.385	3.15	0.9737
		Testing	0.415	4.6	0.9381
	WBC	Training	0.186	1.81	0.9423
		Testing	0.296	3.74	0.9304
	RBC	Training	0.378	2.48	0.9631
		Testing	0.508	3.51	0.9417
	HGB	Training	0.169	2.21	0.9695
		Testing	0.316	2.89	0.921
	HCT	Training	0.306	1.6	0.9643
		Testing	0.342	2.11	0.9429
Danube sturgeon	IT	Training	0.201	4.33	0.9707
		Testing	0.245	5.57	0.9572
	RT	Training	0.361	3.44	0.9498
		Testing	0.406	5.35	0.9305
	WBC	Training	0.336	2.02	0.944
		Testing	0.428	3.78	0.9565
	RBC	Training	0.137	2.64	0.9714
		Testing	0.181	3.75	0.936
	HGB	Training	0.241	3.71	0.9536
		Testing	0.266	5.41	0.9207
	HCT	Training	0.219	2.01	0.9706
		Testing	0.318	3.56	0.9217
Rainbow trout	IT	Training	0.399	3.27	0.9568
		Testing	0.548	4.38	0.9263
	RT	Training	0.171	2.72	0.9657
		Testing	0.212	4.71	0.9583
	WBC	Training	0.329	4.3	0.9466
		Testing	0.355	5.2	0.9267
	RBC	Training	0.253	3.27	0.9669
		Testing	0.298	4.27	0.9239
	HGB	Training	0.265	3.54	0.9665
		Testing	0.352	4.44	0.924
	HCT	Training	0.182	4.49	0.9627
		Testing	0.228	5.57	0.9556

Among the evaluated parameters, the IT and RT predictions for Rainbow trout showed relatively higher RMSE values during testing (0.548 and 0.212, respectively), though still within acceptable limits (*R*^2^ = 0.9263 and 0.9583). In contrast, parameters such as RBC and HGB showed consistent and robust performance across all species, particularly in Danube sturgeon, where testing *R*^2^ values reached up to 0.936 and 0.9207, respectively.

Overall, the ANN models demonstrated their potential as effective tools for estimating physiological responses in fish under anesthetic exposure, supporting their future application in aquaculture research and welfare monitoring.

## 4 Discussion

The findings demonstrated that ANNs possess a high capacity for prediction accuracy in such biological systems, and that interspecies physiological sensitivity differences were directly reflected in the model structures. Although ANN-based approaches have occasionally been applied in human studies to monitor the depth of anesthesia (38), to the best of our knowledge, no machine learning applications have been conducted for the determination of fish anesthetic dose and procedure. However, previous research has suggested that AI-supported systems can provide a new paradigm beyond conventional histological methods in evaluating fish welfare and health (39).

Among the behavioral parameters, the most successful modeling for IT was achieved in rainbow trout, using a tansig–purelin activation function and a 1-8-1 architecture, which yielded stable and low error rates. IT predictions for common carp also demonstrated high accuracy, whereas in Danube sturgeon, although a logarithmic activation function was employed, model stability was limited due to high weight and bias values. For RT, both common carp and rainbow trout achieved similarly successful model performances, particularly with purelin-based activation functions that facilitated stable learning. In Danube sturgeon, model performance for RT was comparatively lower, which may reflect species-specific sensitivity patterns to the anesthetic agent. For an anesthetic agent to be considered applicable, it is expected to meet key criteria, such as being non-toxic to animals, posing no risk to human health, and providing appropriate induction and recovery times (40). In this context, accurate prediction of the anesthetic concentration required to achieve target IT and RT is essential for minimizing stress in fish and ensuring efficient procedural outcomes. Prolonged induction times can increase handling stress and complicate farm operations (41). However, actual economic losses are more likely to occur when fish are exposed to anesthetic agents beyond the required duration or at excessively high concentrations, which may result in mortality (30, 42). The ANN models developed in the current study allow for the estimation of the required concentration of nutmeg oil for achieving the desired IT and RT values in all three fish species. This also enables determination of an appropriate anesthetic dose—defined here as a maximum of 180 s for IT and 300 s for RT—without the need for further *in vivo* experimentation (1). Since IT and RT vary with the physiological response of fish (42, 43), the study aimed to construct species-specific target models. Consequently, the effective anesthetic concentrations for fish can vary widely across species (12).

Hematological indicators are among the most rapid biological markers reflecting fish responses to environmental toxic substances (44). Improperly adjusted doses of anesthetic agents can result in toxic effects (45), and various alterations in blood parameters have been observed in fish subjected to anesthesia. Therefore, it is essential to establish species-specific reference ranges for hematological parameters in fish. In this context, reference ranges for the hematological parameters of common carp, Danube sturgeon, and rainbow trout used in the present study have previously been reported (46–48). Among the hematological parameters, the most consistent results were obtained in the prediction of WBC (leukocyte) levels. For all three species, models using purelin–purelin activation functions and feed-forward network structures achieved highly accurate predictions with minimal error values. This indicates that WBC levels exhibit more predictable and regular responses to varying anesthetic concentrations. WBCs are circulating immune cells that participate in all cell-specific immune responses (3, 49). These cells play a critical role in protecting the fish's body against infections, parasites, toxic substances, and other pathogens (50). An increase in WBC count suggests an activated defense system responding to a perceived threat (51), whereas a decrease implies immunosuppression and increased vulnerability to disease (52). Hence, WBC is considered a key parameter for assessing both stress and immune status in fish. During anesthesia, WBC levels typically increase and later return to baseline (53). This fluctuation reflects a stress response in the fish, which can lead to malfunctions in the hematopoietic system (54). The ability to predict the peak WBC response and its return to normal levels makes WBC a valuable parameter for modeling. Although a model has previously been developed to quantitatively assess fish welfare (55), no comparable model has been established for any anesthetic agent applied to fish.

In the current study, the most accurate predictions for RBC were obtained in common carp, where a robust model was developed using the tansig activation function and a 1-7-1 network architecture. In contrast, the logsig–purelin-based model developed for rainbow trout exhibited relatively lower prediction performance. RBCs are cells responsible for oxygen transport and contain hemoglobin. These cells deliver oxygen to the fish's tissues and remove carbon dioxide (56). Therefore, RBC count provides important insights into the fish's overall physiological condition, oxygen demand, and responses to environmental factors (57). RBC levels may increase in response to hypoxia, elevated temperatures, or high stocking density (58). During anesthesia, opercular movements in fish slow down, which can lead to hypoxic conditions (59). For this reason, modeling RBC values is crucial to distinguish whether the driving force behind changes in oxygen transport is due to the efficacy of the anesthetic agent or secondary hypoxia. In this context, the use of ANN modeling allows for the prediction of RBC fluctuations based on anesthetic concentrations, enabling a data-driven understanding of how oxygen transport efficiency is influenced by anesthesia across species. This predictive capability provides a valuable non-invasive tool for monitoring fish physiological status and optimizing anesthetic protocols accordingly.

In terms of predicting HGB levels, the most successful model was developed for rainbow trout using tansig–purelin activation functions and a 1-10-1 architecture, which, despite its complexity, achieved high accuracy. For common carp, a more compact network architecture also yielded highly accurate results. Lastly, for the HCT parameter, high model performance was achieved across all species, with particularly low error rates in Danube sturgeon and rainbow trout. This indicates that hematocrit levels exhibit less interspecies variability following anesthetic exposure and can be reliably modeled using ANN. HGB is a protein found within erythrocytes that plays a key role in transporting oxygen and carbon dioxide (60), while HCT is a proportional parameter representing the percentage of total blood volume occupied by erythrocytes (61). Both parameters are closely associated with RBC count and typically increase or decrease in parallel with RBC levels (62). Therefore, HGB and HCT values are generally influenced by the same physiological and pathological factors as RBC (63).

While this study focused on three freshwater fish species and a single anesthetic agent (nutmeg oil), the ANN modeling approach used here has potential for broader applications. Given that the input and output structure (e.g., anesthetic dose → IT, RT, hematological response) is modular, similar models could be developed for other species or anesthetic compounds with appropriate data. However, interspecies physiological variability necessitates species-specific training data to ensure accuracy. Therefore, while the network architectures used in this study are transferable in principle, successful application to new contexts would require data-driven re-optimization and validation. This adaptability highlights the potential of ANN-based frameworks as scalable decision-support tools in aquatic anesthesia research and management.

Beyond the laboratory findings, the predictive modeling approach used in this study holds significant promise for field applications in aquaculture. The ability to estimate species-specific anesthetic concentrations based on behavioral and hematological indicators can support the development of anesthesia protocols that minimize stress and mortality during handling, grading, vaccination, or live fish transport. For instance, by targeting optimal induction and recovery times, farm operators can use these models to determine effective doses of nutmeg oil that ensure both fish welfare and operational efficiency without the need for repeated *in vivo* trials. As such, the ANN models presented here may serve as practical decision-support tools for safer and more controlled sedation in diverse aquaculture settings.

Although induction and recovery times were modeled successfully, no LD50 values or detailed toxicity thresholds for nutmeg oil have been reported in fish species to date. As such, the present study focused on non-lethal behavioral and hematological endpoints. Future studies determining lethal or sublethal concentrations would help expand the applicability of modeling approaches in safety-sensitive field practices.

## 5 Limitations

This study has several limitations that should be acknowledged. First, the relatively small sample size raises concerns regarding the robustness and generalizability of the ANN models. While the models demonstrated promising predictive ability, their performance should be interpreted cautiously and validated with larger datasets in future research. Second, the hematological parameters considered are indicators of fish health status. Therefore, the hematological predictions made in this study are valid only within the context of healthy individuals and the tested anesthetic concentration ranges in the referenced datasets. Application to diseased or environmentally stressed fish may not yield accurate predictions.

Finally, since data were obtained from previously published studies, variations in experimental protocols, measurement techniques, and fish handling may have introduced additional variability not fully accounted for by the models. These factors limit the direct extrapolation of the results to broader aquaculture settings. Due to the structure of the ANN models, which yielded a single predictive output per species and parameter based on a given anesthetic concentration, it was not possible to perform statistical analyses such as variance testing or residual distribution plots. This limitation restricts quantitative comparisons across species and should be considered when interpreting interspecies differences in model performance. One limitation of the present study is the lack of integrated toxicity data. While the modeled concentrations were based on previously established non-lethal ranges, the ANN models did not include mortality or sublethal toxicity parameters. Future studies incorporating both behavioral and toxicological endpoints could further refine the safety margins for anesthetic use in aquaculture.

## 6 Conclusion

Overall, species-specific differences in sensitivity to nutmeg oil were observed in terms of induction and recovery times, and these variations were particularly reflected in the complexity of network architectures and the selected activation functions. In contrast, certain hematological variables—especially WBC and HGB—were predicted with high accuracy regardless of the network type employed. The use of feed-forward ANN architectures in combination with various learning algorithms has proven to be an effective approach for multidimensional data analysis in experimental biology. This comprehensive modeling study not only provides a scientific basis for developing species-specific anesthetic protocols but also highlights the potential of AI-based methodologies for improving fish welfare in aquaculture and experimental research. Future studies are encouraged to increase the sample size, compare different anesthetic agents, and develop hybrid models incorporating multiple algorithms. Such advancements are expected to enhance the predictability of biological systems and contribute to more scientifically robust and ethically sustainable practices.

## Data Availability

The raw data supporting the conclusions of this article will be made available by the authors, without undue reservation.
